# A scoping review of equity-focused implementation theories, models and frameworks in healthcare and their application in addressing ethnicity-related health inequities

**DOI:** 10.1186/s13012-023-01304-0

**Published:** 2023-10-16

**Authors:** Papillon Gustafson, Yasmin Abdul Aziz, Michelle Lambert, Karen Bartholomew, Nicole Rankin, Adam Fusheini, Rachel Brown, Peter Carswell, Mihi Ratima, Patricia Priest, Sue Crengle

**Affiliations:** 1 Ngāi Tahu Māori Health Research Unit, Division of Health Sciences, University of Otago, Dunedin Campus, Dunedin, Aotearoa PO Box 56, 9054 New Zealand; 2 Te Whatu Ora Waitematā and Te Toka Tumai Auckland, Auckland, Aotearoa New Zealand; 3 Evaluation and Implementation Science Unit, Melbourne School of Population and Global Health, University of Melbourne, Melbourne, Australia; 4 Preventive and Social Medicine, University of Otago, Dunedin Campus, Dunedin, Aotearoa New Zealand; 5 National Hauora Coalition, Auckland, Aotearoa New Zealand; 6 Synergia Ltd, Auckland, Aotearoa New Zealand; 7 Taumata Associates, Hāwera, Aotearoa New Zealand

**Keywords:** Health inequity, Implementation, Theories, Models, Frameworks, Ethnic inequities, Scoping review

## Abstract

**Background:**

Inequities in implementation contribute to the unequal benefit of health interventions between groups of people with differing levels of advantage in society. Implementation science theories, models and frameworks (TMFs) provide a theoretical basis for understanding the multi-level factors that influence implementation outcomes and are used to guide implementation processes. This study aimed to identify and analyse TMFs that have an equity focus or have been used to implement interventions in populations who experience ethnicity or ‘race’-related health inequities.

**Methods:**

A scoping review was conducted to identify the relevant literature published from January 2011 to April 2022 by searching electronic databases (MEDLINE and CINAHL), the Dissemination and Implementation model database, hand-searching key journals and searching the reference lists and citations of studies that met the inclusion criteria. Titles, abstracts and full-text articles were screened independently by at least two researchers. Data were extracted from studies meeting the inclusion criteria, including the study characteristics, TMF description and operationalisation. TMFs were categorised as determinant frameworks, classic theories, implementation theories, process models and evaluation frameworks according to their overarching aim and described with respect to how equity and system-level factors influencing implementation were incorporated.

**Results:**

Database searches yielded 610 results, 70 of which were eligible for full-text review, and 18 met the inclusion criteria. A further eight publications were identified from additional sources. In total, 26 papers describing 15 TMFs and their operationalisation were included. Categorisation resulted in four determinant frameworks, one implementation theory, six process models and three evaluation frameworks. One framework included elements of determinant, process and evaluation TMFs and was therefore classified as a ‘hybrid’ framework. TMFs varied in their equity and systems focus. Twelve TMFs had an equity focus and three were established TMFs applied in an equity context. All TMFs at least partially considered systems-level factors, with five fully considering macro-, meso- and micro-level influences on equity and implementation.

**Conclusions:**

This scoping review identifies and summarises the implementation science TMFs available to support equity-focused implementation. This review may be used as a resource to guide TMF selection and illustrate how TMFs have been utilised in equity-focused implementation activities.

**Supplementary Information:**

The online version contains supplementary material available at 10.1186/s13012-023-01304-0.

Contributions to the literature

- Implementation science is recognised as an approach to address health inequities and there is a growing number of theories, models and frameworks (TMFs) available to support equity-focused implementation activities.
- This scoping review identifies and describes equity-focused implementation science TMFs used in healthcare with respect to their purpose, components and utilisation, providing a resource to support health researchers, clinicians, funders and other decision-makers in selecting a TMF to guide equity-focused implementation projects.
- The review summarises how equity and systems-level factors influencing implementation outcomes (micro-, meso- and macro-level factors) are represented in different TMFs.

## Introduction

Health inequities, which are differences in health between groups of people that are unnecessary, avoidable, unfair and unjust, are well documented globally [1–6]. Despite increased policy and research efforts over the past 30 years, people continue to experience differences in health based on social, economic, demographic and geographic factors [7–9]. Health inequities are the manifestation of complex historical and contemporary political, legal, social, economic and institutional processes, structures and policies that result in unequal power and resource distribution in society [8, 10, 11].

Ethnicity or ‘race’-related health inequities are pervasive and are an important policy focus in many jursidictions, including the USA [12], UK [13], Canada [14], Australia [15] and New Zealand [16]. Ethnic health inequities are health inequities experienced by groups of people where the group is defined by shared geographic origin and ancestry—often accompanied by shared history, language, beliefs and customs [17]. It should be noted that while the authors recognise that groupings based on ‘race’ remains commonplace in some countries, this term is rooted in beliefs about inherent biological differences between groups of people based on physical characteristics for which there is no scientific evidence (e.g. skin colour, facial features or hair texture) [17]. Ethnicity is therefore used in this paper to refer to groups of people with a shared geographic ancestry and encompasses groups which may be categorised as ‘races’. Minoritised ethnic groups have less access to the social determinants of health, health services, quality and culturally appropriate care, resulting in poorer health outcomes that include a lower life expectancy, and increased incidence of, and mortality from, communicable and non-communicable diseases [3, 18–25]. A 2016 population study of 28 Indigenous and tribal populations in 23 countries found poorer outcomes compared to non-Indigenous populations across a range of health and social measures [3]. These inequities reflect the historic and ongoing impacts of colonisation [26].

Inequities in implementation are increasingly recognised as an important factor in the unequal benefit of health interventions between groups of people who experience differing levels of advantage [27, 28]. Evidence-based interventions or practices often have limited uptake and sustainability when implemented in disadvantaged populations [28, 29]. Furthermore, minoritised populations are under-represented in research, meaning the evidence base for interventions or practices that are appropriate and effective is limited [28, 30]. Various causes are attributed to this, including lack of attention by investigators, lack of resources and dedicated strategies for target populations (including health literacy and culturally or language-appropriate material), exclusion criteria in clinical trials, use of culturally inappropriate research methods, and mistrust by the minoritised populations about participating in research [30, 31]. For minoritised ethnic groups, racism is a key determinant of health equity and contributes to the under-representation of these groups in research [32, 33]. There have been recent calls to the implementation science field to explicitly address structural racism [34, 35].

Identifying the sources and sites of inequity and addressing these through intervention and implementation pathway design are crucial to achieving equitable health outcomes [27]. The field of implementation science, which studies the translation of evidence-based research findings and practices into routine practice, provides a methodological approach to systematically explore what is being delivered and to whom, under what conditions and what changes are required to meet the target population’s needs [27, 36–41]. Theories, models and frameworks (TMFs) are used in implementation science to provide a theoretical basis for understanding implementation, including the factors that influence implementation outcomes, and to guide the process of implementation [42]. Nilsen provides a classifying taxonomy for implementation science TMFs according to three broad aims: (1) describing and/or guiding the process of translating research into practice (process models), (2) understanding or explaining factors influencing implementation outcomes (determinant frameworks, classic theories and implementation theories), and (3) evaluating implementation (evaluation frameworks) [42]. In recent years, a number of implementation TMFs have been developed or adapted with an explicit equity focus [36, 43]. These, to the best of our knowledge, have not been systematically identified and described.

Interventions to address health inequities have often targeted patients as the unit of change, e.g. education to increase knowledge and change behaviour [44]. However, inequity is a systemic issue, and resolution, therefore, requires a systems approach. Systems thinking approaches problem-solving by considering the dynamic system in which the ‘problem’ is situated, seeking to understand the relationships, interactions, perspectives and behaviours of the components that affect the system [45, 46]. Viewing health inequities through a systems thinking lens facilitates understanding and transformation of the system that generates and replicates these inequities by shifting from a health outcomes focus to a broader focus on the entire system in which health and well-being are embedded [47]. This multi-level view allows for inequities at each level (individual, interpersonal, organisational, community and societal) to be appropriately and effectively addressed through implementation pathway design and delivery [46].

This scoping review explores the literature relating to equity-focused implementation science TMFs, with a particular focus on how these have been, or may be, used to address ethnic health inequities. The specific objectives were to (1) identify TMFs that have an equity focus or have been used to implement interventions in populations who experience ethnicity-related health inequities and (2) analyse the TMFs with respect to their purpose, components, how equity and system-level factors influencing implementation are incorporated, and operationalisation (i.e. how the TMF had been used in the implementation of an intervention). The scoping review methodology was determined to be the most suitable to review this literature as it allows exploration of the extent, variety and characteristics of evidence, including mapping key concepts and identifying research gaps, from diverse sources to address a research question [48–51]. Unlike a systematic review, quality assessment is not required and a broader range of sources (e.g. grey literature) can be included [51].

## Methods

### Protocol design

A protocol for this scoping review was published previously [52]. The protocol design was informed by the six-stage methodological framework for scoping reviews developed by Arksey and O’Malley [48] and extended by Levac and colleagues [53]. The Preferred Reporting Items for Systematic Reviews and Meta-Analyses extension for Scoping Review (PRISMA-ScR) checklist was used to guide the reporting of the results of this review (Additional file 1) [51]. The study protocol includes a second review question (what implementation factors aid or inhibit the achievement of equity in health interventions? [52]), the results of which will be published separately. This was a pragmatic decision due to the volume of results and analysis associated with each research question.

### Identifying the research question

The research question was developed collaboratively through consultation with the research team to guide the search strategy: What equity TMFs have been developed to inform the design and implementation of interventions in the health sector?

For this study, an intervention was defined as ‘any activity undertaken with the objective of improving human health by preventing disease, by curing or reducing the severity or duration of an existing disease, or by restoring function lost through disease or injury’ (p.41–42) [5]. This included what Brown et al. broadly describe as the ‘7 Ps’: programmes, practices, principles, procedures, products, pills and policies [54].

### Identifying the relevant studies

Literature searching was conducted in three phases: (1) electronic database searching, (2) hand-searching of key journals, and (3) searching the reference lists and citations of studies meeting the inclusion criteria. The database, reference list and citation searches were limited to 2011 to ensure good coverage of the equity-focused implementation science literature, which has increased markedly in the past 5 years.

1. The electronic databases MEDLINE (Ovid) and CINAHL were searched to identify literature published between 1 January 2011 to the present (final search executed 5 April 2022; search strategy and results in Additional file 2). These databases were selected as they were determined by the research team to provide the best coverage of the relevant biomedical literature. The Dissemination and Implementation (D&I) model database was also searched to identify implementation science TMFs with a health equity focus (final search executed on 5 April 2022; search strategy and results in Additional file 2).
2. Five key journals were hand-searched for articles relevant to the research question: BMC Health Services Research, Implementation Science, Implementation Science Communications, Implementation Research and Practice, and International Journal for Equity in Health. These journals were identified by reviewing the database search results and from recommendations by the research team. Due to the large number of results and associated time constraints with reviewing these, the search was limited to 2015 to 2021, rather than starting in 2011 as outlined in the scoping review protocol [52].
3. Once the first three phases of searching were completed and the eligible papers were identified, the reference lists of these studies were searched to identify any additional relevant literature relating to the research question. In addition, an overview article on health equity in implementation science [36] was searched for other references describing equity-focused TMFs. Finally, the citations of novel or adapted TMFs were searched in Google Scholar (using the ‘cited by’ function) to identify additional publications where the TMF had been operationalised.

As described in the scoping review protocol, a grey literature search limited to the Aotearoa New Zealand context was also undertaken as part of this review (Additional file 3). However, the results have not been included in this analysis.

### Study selection

Preliminary inclusion and exclusion criteria were developed from the research question and piloted on twenty titles and abstracts by three members of the research team (PG, YAA and ML). All titles and abstracts were then screened independently by two researchers (PG and YAA) to assess alignment with the aims of the scoping review and papers that were ineligible were excluded. A third researcher (ML) was consulted when consensus could not be reached. The inclusion and exclusion criteria were reviewed and refined in consultation with the lead researcher (SC) before proceeding to full-text review (Table 1). The full text of potentially eligible studies was independently reviewed against the inclusion and exclusion criteria by two researchers (PG and YAA); any disagreements were resolved through discussion with a third researcher (ML). Title/abstract and full-text screening outcomes and reasons for inclusion/exclusion were documented using Microsoft Excel Version 2209.

**Table 1:** Inclusion and exclusion criteria for the scoping review

Inclusion criteria	Exclusion criteria
1. English language	1. Non-English language
2. Published between January 2011 to the present (or January 2015 to the present for articles identified by hand-searching key journals)	2. Full text unavailable
	3. Published prior to 2011 (or 2015 for articles identified by hand-searching key journals)
	4. Commentaries, discussion or working papers, policy documents, editorials, opinions and letters, conference proceedings, quantitative research that does not meet the inclusion criteria
3. Full text available	
4. Describe an equity-focused implementation science TMF, i.e. equity (or related terms like disparity or inequality) is explicitly mentioned or equity (or parity/equality) is an explicit aim of the TMF, with or without operationalisation in intervention implementation	
	5. Studies describing TMFs from fields other than implementation science, e.g. health equity frameworks
	6. Studies describing interventions targeting non-ethnicity-related health inequities
5. Describe the use of an established implementation science TMF to implement an intervention in an Indigenous or other minoritised ethnic/’racial’ group	7. Studies in non-healthcare settings (e.g. schools, churches) without health provider involvement
	8. Interventions that do not address a particular health need
	9. Study protocols that lacked sufficient detail about the TMF
6. Interventions conducted in healthcare settings; this included community-based health interventions if there was health provider involvement	

### Charting the data

Two researchers (PG and YAA) extracted data as described in the scoping review protocol [52]. This included information about (1) study characteristics (title, author, year published, geographical region, target population, setting and study category), (2) TMF description, (3) TMF development, (4) TMF components, and (5) application/operationalisation of TMF (study demographics, setting, methodology, relevant outcomes).

### Collating, summarising and reporting the results

Using the extracted data, the equity-focused implementation science TMFs were categorised according to the classification outlined by Nilsen [42], which describes five types of TMFs that align with three main aims (definitions provided in Additional file 4: Table S1). We acknowledge that these categories are not fixed and TMFs can belong to more than one category and be used for more than one purpose [55]. Our categorisation, therefore, reflects which classification the TMF is most consistent with, or how it was previously classified by Nilsen [42], rather than conveying an exclusive categorisation or purpose.

TMFs within each category were described with respect to their purpose, components, how equity and system-level factors influencing implementation were incorporated, and operationalisation (i.e. how the TMF had been used in the implementation of an intervention). The equity focus of TMFs was classified as ‘explicit’ if terms related to equity (inequity, parity/disparity, equality/inequality) were mentioned in the TMF either as a stated aim or at the dimension or construct level. The equity focus was considered ‘implicit’ if the context of TMF development was to address a particular health equity need through detecting, understanding or reducing health inequities [56]. If the TMF did not incorporate an explicit or implicit health equity focus but had been applied in an equity context, i.e. implementing an intervention in a population experiencing ethnic health inequities, then the equity focus was classified as ‘applied’. System factors were categorised as micro-level (factors associated with individuals), meso-level (factors associated with communities, organisations and/or services), and macro-level (factors external to the organisation, community or service, such as policy). Systems thinking was deemed ‘fully considered’ if multi-level factors were explicitly described in the TMF or ‘partially considered’ if systems-level factors were either partly represented or were not explicitly described but the wording was such that it would allow for, or prompt, user interpretation to consider systems factors. All TMFs at least partially considered or represented systems factors.

### Consultation

Stakeholder and expert consultations were undertaken as described in the scoping review protocol [52]. Briefly, the research team, with expertise in health equity, Māori (the Indigenous peoples of New Zealand) health and implementation science, and the Kāhui (advisory group) comprised experts in Māori health research and service provision, Iwi (tribe) representatives and health service consumers, reviewed the findings to identify any gaps and provide feedback based on their knowledge of the international and local literature relating to Indigenous health inequities.

## Results

### Search results

**Fig. 1:**
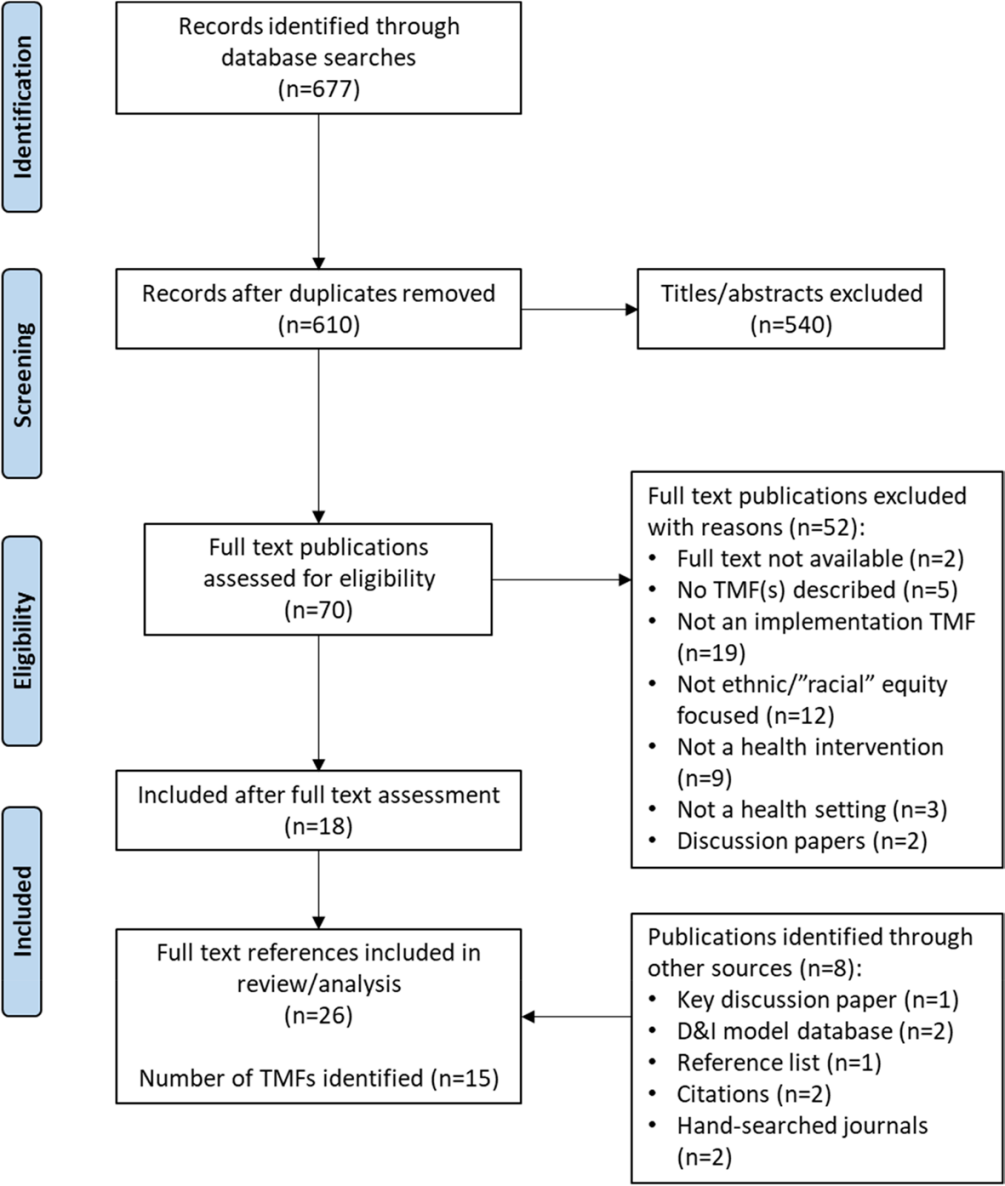
PRISMA flow diagram of the literature search and publication selection

The MEDLINE and CINAHL database searches yielded 610 unique results (after duplicates were removed). After screening titles and abstracts, 70 publications were eligible for full-text review. Following full-text review, 18 publications met the inclusion criteria, identifying 11 TMFs. A further eight publications meeting inclusion criteria were identified from (1) the reference list of studies identified through the database search that met the inclusion criteria, (2) the D&I model database, (3) reference list searching of one key overview article on health equity and implementation science [36], (4) hand-searching key journals, and (5) forward searching the citations of studies that meet the inclusion criteria. An additional four TMFs were identified from these sources. In total, 26 papers describing 15 TMFs and their operationalisation were identified for inclusion in this scoping review (Fig. 1).

### Description and classification of TMFs

The identified TMFs and their application in health intervention implementation are summarised in Table 2. Nine of the TMFs were novel and had an equity focus [57–65], three were equity-focused adaptations of established TMFs [11, 66, 67], and three were established TMFs applied in an equity context [68–74]. Five of the novel TMFs were developed in the USA [57, 60, 63, 64, 66]: one each in Australia [62], Aotearoa New Zealand [61], Latin America [58], Canada and Tanzania [59]. The most common novel TMF to be operationalised was the He Pikinga Waiora (HPW) Implementation Framework (three studies) [75–77]. The Consolidated Framework for Implementation Research (CFIR) was the most commonly operationalised established TMF [69–71]. Four TMFs (novel or adapted with an equity focus) had not been operationalised [11, 57, 62, 63].

**Table 2:** Summary of TMFs and their operationalisation

Name	Author	Origin/location	Purpose	Type	Description	Equity focus* (explicit/implicit/applied)	Systems focus	Operationalisation
**Determinant frameworks**								
Consolidated Framework for Implementation Research (CFIR)	Gordon et al. [68], Gordon et al. [69], Goff et al. [70], Lam et al. [71]	All USA	To identify facilitators and barriers to implementation	Established [79]	Five domains of factors that determine implementation success: (1) Intervention characteristics; (2) Outer setting; (3) Inner setting; (4) Characteristics of individuals; (5) Process	Applied	Fully considered Micro-level: Characteristics of Individuals Meso-level: Inner setting Macro-level: Outer setting	Culturally appropriate kidney transplant programme for Hispanic people [68, 69]. CFIR used prior to implementation to identify key stakeholders’ perceptions of the facilitators and barriers to implementation. Post-partum depression screening protocol delivered in practices that serve minoritised ethnic and racial groups [70]. CFIR used prior to implementation to identify perceived facilitators and barriers to screening and referral. Interventions to increase colorectal cancer screening in clinics serving predominately (> 82%) minoritised ethnic and racial groups [71]. CFIR used post-implementation for evaluation.
Health Equity Implementation Framework (HEIF)	Woodward et al. [66]	USA	To identify health equity determinants so that interventions and implementation strategies can be tailored or adapted to advance health equity	Adapted	Five domains of factors influencing implementation outcomes and health equity: (1) Characteristics of the Innovation; (2) Clinical Encounter; (3) Patient & Provider Factors; (4) Inner & Outer Context; (5) Societal Influence	Explicit Identifies factors that explain the causes of health equities across multiple levels (patients, providers, clinical encounters and the health system)	Fully considered Micro-level: Clinical Encounter and Patient and Provider Factors Meso-level: Inner context Macro-level: Outer context, Societal influence	Hepatitis C treatment in Black veterans [66]. HEIF used to explore patient-identified barriers and facilitators to treatment. Social needs screening survey in a primary care clinic serving predominantly minoritised ethnic and racial groups [80]. HEIF used to identify clinician and patient perspectives on facilitators and barriers to implementing the screening survey. Adapted for use in the field of paediatric rheumatology where inequities in patient care and health outcomes are evident [81]. This adaptation retains the same five overarching domains of HEIF with a focus in the long-term nature of caring for paediatric rheumatology patients.
Integrated Practical, Robust Implementation and Sustainability Model (PRISM) and Socio-Ecological Model (SEM) framework	Henderson et al. [65]	USA	To guide development and implementation of a breast screening programme	Novel	Integrated framework combining PRISM (an implementation science framework) and SEM (a behavioural health framework)	Implicit Framework developed to guide design and implementation of a screening and navigation programme to address breast cancer disparities	Fully considered Micro-level: Patient perspectives and characteristics (PRISM); individual and interpersonal levels (SEM) Meso-level: Organisational perspectives and characteristics, Implementation and Sustainability Infrastructure and External Environment (PRISM); Community and Organisation levels (SEM) Macro-level: policy level (SEM)	Breast cancer screening programme (Mi-MAMO) for underserved populations (58% Hispanic/Latina, 34% non-Hispanic Black) [65]. Programme design and implementation addressed factors affecting breast cancer screening, early detection and treatment that were identified across the different levels of the integrated framework.
He Pikinga Waiora (HPW) Implementation Framework	Oetzel et al. [61]	Aotearoa New Zealand	Provide a theoretical foundation and guide for designing and implementing effective and culturally-appropriate interventions for communities experiencing health inequities	Novel	Comprises four elements: Community Engagement; Cultural Centredness; Systems Thinking; Integrated Knowledge Translation. These elements are underpinned by a Kaupapa Māori approach, which prioritises indigenous history, development and aspirations.	Explicit Each element included has been identified as important for advancing Indigenous health equity	Fully considered Captured primarily in the Systems Thinking element Micro-level: Cultural Centeredness Meso-level: Community Engagement and Integrated Knowledge Translation	Retrospective analysis of lifestyle interventions for diabetes prevention in Indigenous communities in Australia, Canada, New Zealand and the USA [61]. Co-design of lifestyle interventions for Māori communities in New Zealand [75, 76]. Evaluation of two researcher-community partnerships that were engaged to co-deign and implementation of lifestyle interventions for diabetes prevention [77].
**Implementation theories**								
Capability, Opportunity, Motivation and Behaviour (COM-B)	Handley et al. [72], Gould et al. [73]	USA, Australia	Model for understanding behaviour; used as a basis for designing interventions that aim to change behaviour	Established [82]	COM-B helps to identify possible behavioural targets for interventions across three domains: Capability, Opportunity and Motivation. Forms part of the Behaviour Change Wheel	Applied	Partially considered Micro-level: Motivation The Opportunity domain could include consideration of meso- and/or macro-factors influencing behaviours	Health IT coaching and resource programme for Latina women with recent gestational diabetes [72]. COM-B used to analyse data relating to barriers and facilitators for post-partum women. engaging with diabetes prevention behaviours Smoking cessation service for pregnant Indigenous Australian women [73]. COM-B was used to inform intervention design. N.B. This study also used the TDF (a determinant framework) to provide examples of other domains that aligned with the COM-B model.
**Process models**								
Equity-based Framework for Implementation Research (EquIR)	Eslava-Schmalbach et al. [58]	Latin America	Reduce or prevent the increase of existing inequalities during the implementation of equity-focused health programs, policies or interventions	Novel	Five steps: (1) Population's health status; (2) Planning the programme; (3) Designing equity-focused implementation research; (4) Implementing equity-focused implementation research; (5) Equity focused implementation outcomes	Explicit Each step of the framework has an equity lens applied. Includes gathering data on inequities, identifying strategies to reduce inequities, involving key stakeholders, identifying facilitators and barriers to implementation and equity-focused evaluation.	Partially considered Multi-level systems factors are not explicitly described, but the framework prompts users to identify facilitators and barriers to implementation, and design strategies to overcome these, which, depending on the intervention, could include micro-, meso- and macro-factors	Implementation of a programme in a population of disadvantaged children in Bolivia [58]. An equity lens was provided to the programme using the EquIR. Steps were revised based on equity considerations.
Intervention and Research Readiness Engagement and Assessment of Community Health Care (I-RREACH) tool	Maar et al. [59]	Canada, Tanzania	Guide implementation of interventions in low-resource settings to ensure the intended health benefits are achieved	Novel	The tool includes three phases: (1) A community fact sheet to determine if the characteristics are suitable for implementation of the intervention; (2) A key informant interview guide to gather practical information on what is required for successful implementation; (3) A focus group interview guide to gather information on the lived experience of the intended recipients of the intervention	Implicit Guides the process of identifying factors that influence implementation in low-resource settings (low- and middle-income countries and disadvantaged populations in high-income countries)	Partially considered Micro-level: Perceptions of key informants and community members about implementation Meso-level: gathers information on a range of meso-level factors relevant to the implementation context	Text messaging service to encourage blood pressure measurement and feedback between patients and health providers [59]. The tool was developed through participatory research prior to implementation of the trial.
Transcreation Framework	Nápoles and Stewart, [60]	USA	Step-by-step guide to intervention design and implementation to reduce health disparities, in partnership with the target community	Novel	Seven-step process for implementation: (1) Identify community infrastructure and engage partners; (2) Specify theory; (3) Identify multiple inputs for new programme; (4) Design intervention prototype; (5) Design study, methods and measures for community setting; (6) Build community capacity for delivery; (7) Deliver ‘transcreated’ intervention and evaluate implementation processes	Explicit Focuses on community partnership to reduce inequities in intervention adaptation and delivery	Partially considered Primarily focuses on the meso-level, i.e. the community setting and its capacity for the intervention	Development of a stress management intervention for Latina cancer survivors [83]. The framework facilitated community engagement and programme adaptation, enabling recruitment goals to be exceeded and the intervention to be implemented successfully.
Indigenous Health Promotion Tool Implementation Model	Percival et al. [62]	Australia	Provide a framework for Indigenous health promotion tool implementation planning, documentation and evaluation	Novel	The model describes the: (1) Conditions influencing implementation (Reciprocity, Change agents, Governance and resources); (2) Four processes guiding implementation (Engaging and relating, Developing and using evidence, tailoring for diverse groups, programmes and settings, Strengthening capacity); (3) Benefits (Participant satisfaction and control, Workforce recruitment and capacity, Organisational resources, systems and partnerships, Programme sustainability and spread)	Implicit Each aspect of the model has been identified as important for culturally responsive health promotion in Indigenous peoples	Partially considered Primarily focuses on the micro- and meso-levels Micro-level: Reciprocity and Change agents Meso-level: Governance and resources	Not operationalised
ConNECT Framework	Alcaraz et al. [57]	USA	Link behavioural medicine and health equity science to achieve equitable health outcomes	Novel	Five interrelated principles: (1) Integrating Context; (2) Fostering a Norm of Inclusion; (3) Ensuring Equitable Diffusion of Innovations; (4) Harnessing Communication Technology; (5) Prioritising Specialised Training The principles are applied across the research to practice continuum through the phases of Discovery, Development, Delivery and Dissemination	Explicit All principles have an equity lens applied. Includes appreciating the situational and interactive influences on health, engaging with and examining diverse groups, facilitating equitable intervention dissemination, utilising communication strategies to enhance reach, and workforce development through integrating education, training, and mentoring approaches.	Partially considered Systems level factors are not explicitly described, however, the Integrating Context, Fostering a Norm of Inclusion and Ensuring Equitable Diffusion of Innovations domains could reasonably include consideration of micro-, meso- and macro-factors	Not operationalised
Collaborative Intervention Mapping Framework	Cabassa et al. [64, 84]	USA	Overcome barriers to the modification, pre-implementation, and use of evidence-based approaches in real-world settings by using a collaborative approach	Novel	Combines Community-Based Participatory Research (CBPR) principles (shared health concern, ownership, co-learning and capacity building) with Intervention Mapping (IM). IM comprises six steps: (1) Problem analysis; (2) Review of intervention objectives and theoretical foundations; (3) Modification of intervention methods and strategies; (4) Development of revised intervention; (5) Development of adoption and implementation plan; (6) Evaluation	Implicit Uses CBPR principles to ensure sociocultural and system factors are considered when translating interventions to new contexts	Partially considered Primarily considers factors at the meso-level of influence, i.e. focus on community engagement and modifying the intervention to ensure it is appropriate for the community context	Health care manager intervention (PCARE – care coordination and patient activation) for Hispanic people with serious mental illness [84]. The collaborative framework was used to adapt the intervention to improve its reach in the local community and reduce health disparities.
**Evaluation frameworks**								
Reach, Effectiveness, Adoption, Implementation and Maintenance (RE-AIM)	Glasgow et al. [74]	USA	Plan and evaluate implementation of interventions across five key dimensions	Established [85]	RE-AIM comprises five dimensions: Reach, Effectiveness, Adoption, Implementation, Maintenance	Applied	Partially considered Primarily focuses on the micro- and meso-levels Micro-level: Reach and Effectiveness Meso-level: Adoption, Implementation and Maintenance	Weight loss and hypertension management intervention targeting a high-risk population (70% African-American, 13% Hispanic) [74]. RE-AIM used to identify equity issues across the different domains relating to implementation and dissemination if the intervention.
Reach, Effectiveness, Adoption, Implementation and Maintenance (RE-AIM) extension for sustainability	Shelton et al. [67]	USA	Guide planning, measurement, evaluation and adaptation of evidence-based interventions with a focus on sustainability	Adapted	Extension of the RE-AIM framework to enhance sustainability, by focusing on Dynamic Context and Culture, Health Equity, and Costs, Resources and Capacity across the implementation cycle and RE-AIM domains	Explicit The framework includes a health equity domain that prompts users to consider health equity across each of the RE-AIM domains	Partially considered Micro-level: Reach and Effectiveness Meso-level: Adoption, Implementation and Maintenance Macro-level influences are captured by the Costs, Resources and Capacity domain	Not operationalised
Adaptation of Proctor et al. framework	Baumann and Cabassa, [11]	USA	To illustrate how application of an equity lens can help to integrate the implementation science and health inequities research fields	Adapted	Equity-focused adaptation of Proctor et al.’s implementation outcomes framework [86], focusing on five elements: (1) Focus on reach from the beginning; (2) Design and select interventions for vulnerable populations with implementation in mind; (3) Implement what works and develop implementation strategies that can help reduce inequities in care; (4) Develop the science of adaptation; (5) Use an equity lens for implementation outcomes	Explicit The framework applies an equity lens across each element	Partially considered Emphasises Reach (micro-level) and focusing on the implementation context, which, depending on the intervention, could include micro-, meso- and macro-level factors	Not operationalised
**Hybrid frameworks**								
EQ-DI	Yousefi et al. [63]	USA	To illustrate the interaction between the fields of health equity and D&I science	Novel	Health equity sensitises D&I planning and evaluation frameworks. D&I science operationalises EBIs to promote health equity by providing tools, methods and approaches for planning and evaluation	Explicit Health equity sensitises D&I science by identifying, acknowledging and addressing the conditions in which inequities are created and perpetuated across multiple levels (individuals, relationships, community and system-levels)	Fully considered Prompts users to consider the multiple levels and complex dynamics that influence equitable implementation	Not operationalised

*CBPR* community-based participatory research; *CFIR* Consolidated Framework for Implementation Research; *COM-B* Capability, Opportunity, Motivation and Behaviour; *D&I* Dissemination and Implementation; *EBI* evidence-based intervention; *EquIR* Equity-based Framework for Implementation Research; *HEIF* Health Equity Implementation Framework; *HPW* He Pikinga Waiora; *IM* Intervention Mapping; *iPARIHS* Integrated Promoting Action on Research Implementation in Health Services; *I-RREACH* Intervention and Research Readiness Engagement and Assessment of Community Health Care; *PRISM* Practical, Robust Implementation and Sustainability Model; *SEM* Socio-Ecological Model; *RE-AIM* Reach, Effectiveness, Adoption, Implementation and Maintenance; *TDF* Theoretical Domains Framework

^*^Explicit equity focus: Terms related to equity [inequity, parity/disparity, equality/inequality] are mentioned in the TMF either as an aim or at the dimension or construct level

*Implicit equity focus:* Context of TMF development is to address a particular health equity need through detecting, understanding or reducing health inequities [56]

*Applied equity focus:* TMF does not incorporate an explicit health equity focus but had been applied in an equity context, i.e. implementing an intervention in a population experiencing ethnic health inequities

Each TMF was classified according to the categories described by Nilsen [42], resulting in four determinant frameworks, one implementation theory, six process models and three evaluation frameworks (Table 3). None of the TMFs identified aligned with the classic theory classification. One framework included elements of determinant, process and evaluation TMFs and was, therefore, classified as a ‘hybrid’ framework.

**Table 3:** Equity-focused TMFs classified by type

Classification	TMF
Determinant framework (*n* = 4)	Consolidated Framework for Implementation Research (CFIR) [68–71]
	Health Equity Implementation Framework (HEIF) [66]
	He Pikinga Waiora Implementation Framework (HPW) [61]
	Integrated PRISM and SEM framework [65]
Implementation theory (*n* = 1)	Capability, Opportunity, Motivation and Behaviour (COM-B) model [72, 73]
Process model (*n* = 6)	Equity-based Framework for Implementation Research (EquIR) [58]
	Intervention and Research Readiness Engagement and Assessment of Community Health Care (I-RREACH) [59]
	Transcreation Framework [60]
	Collaborative Intervention Planning Framework [64, 84]
	ConNECT Framework [57]
	Indigenous Health Promotion Tool Implementation Model [62]
Evaluation framework (*n* = 3)	Reach, Effectiveness, Adoption, Implementation and Maintenance (RE-AIM) [74]
	Extension of RE-AIM for sustainability [67]
	Adaptation of Proctor et al. [11]
Hybrid (*n* = 1)	EQ-DI [63]

### Determinant frameworks

Four of the TMFs identified were determinant frameworks (Table 3). Two (CFIR, HEIF) were generic frameworks, identifying a comprehensive range of factors that influence implementation outcomes and were not specific to a particular intervention or population [66, 78]. In contrast, HPW identifies determinants of effective and culturally appropriate implementation for Indigenous communities [61], and the integrated PRISM and SEM framework was developed to guide the development of a specific intervention (that is, a breast screening programme for under-served communities) [65].

Three determinant TMFs (HEIF, HPW, the integrated PRISM and SEM framework) have an equity focus [61, 65, 66]. The HEIF aims to promote health equity by focusing implementation assessments on the determinants of health equity across five domains: Characteristics of the Innovation, Clinical Encounter, Patient and Provider Factors, Inner and Outer Context and Societal Influence [66]. The HEIF has been used to identify the facilitators and barriers to the implementation of interventions in populations experiencing ethnicity-related health inequities [66, 79]. One study reported the adaptation of the HEIF to a specific medical specialty (paediatric rheumatology) to address disparities in patient care and health outcomes [80].

HPW is a co-design framework that aims to improve health outcomes and achieve health equity by facilitating the design and implementation of effective and culturally appropriate interventions for Indigenous communities [61]. The framework describes four elements (determinants): Community Engagement, Cultural Centredness, Integrated Knowledge Translation and Systems Thinking [61]. HPW has been used to guide the co-design process and to evaluate interventions implemented in Māori communities in Aotearoa New Zealand [75–77].

The integrated PRISM and SEM framework describes how different levels of influence on health behaviours and outcomes (from the SEM) apply across the PRISM implementation framework [65]. Individual-, interpersonal- and organisation-level SEM influences include the patient and organisation perspectives on the intervention, recipient (organisation and patient) characteristics and the implementation and sustainability infrastructure from the PRISM framework [65]. The community-level SEM influences include the External Environment and the Implementation and Sustainability Infrastructure [65]. The policy-level factors are those ultimately determining the Reach and Effectiveness of the programme [65]. The integrated framework was developed and used to guide the implementation of a breast cancer screening and patient navigation programme for predominantly minoritised ethnic populations in the USA [65].

The CFIR (original version, first published in 2009) does not have an equity focus but has been utilised in the implementation of interventions in populations who experience ethnicity-related health inequities. The CFIR describes 39 constructs across five domains that determine implementation success: Intervention Characteristics, Outer Setting, Inner Setting, Characteristics of Individuals and Process [78]. Three studies used the CFIR to identify facilitators and barriers to the implementation of health interventions in minoritised ethnic or racial populations in the USA [69–71].

Three TMFs (CFIR, HEIF, integrated PRISM and SEM framework) provide comprehensive consideration of micro-, meso- and macro-level influences on implementation outcomes (Table 2). The micro-level of influence is represented at the domain level in these TMFs as the Characteristics of Individuals (CFIR [78]), patient characteristics and perspectives (integrated PRISM and SEM model [65]) and Patient and Provider Factors (HEIF [66]). The meso-level of influence is represented as the Inner Context (CFIR and HEIF [66, 78]) and the Organisational perspectives and characteristics, External Environment and Implementation and Sustainability Infrastructure domains (integrated PRISM and SEM model [65]). The macro-level of influence is represented by the Outer Context domain (CFIR and HEIF [66, 78]), the Societal Influence domain (HEIF [66]) and the Policy level of the SEM (integrated PRISM and SEM model [65]).

HPW is less comprehensive than these other determinant TMFs as it focuses specifically on implementation effectiveness and appropriateness in Indigenous communities, rather than the broad range of implementation determinants [61]. Within this context, however, the HPW framework Systems Thinking element asks users to consider a range of perspectives, levels and understandings when implementing interventions [61].

### Implementation theories

One TMF was an implementation theory (Table 3). The COM-B model describes the behavioural aspects of implementation across three domains: Capability, Opportunity and Motivation [72]. COM-B is not equity-focused but has been used to adapt a gestational diabetes programme for Latina women [72] and to design a smoking cessation service for Aboriginal and Torres Straight Islander people in Australia [73]. COM-B is primarily focused on individual-level behaviours. However, the Opportunity domain, which is the physical and social factors influencing behaviour, could include consideration of meso- or -macro-level factors depending on the implementation context.

### Process models

Six TMFs were process models (Table 3). Five are generic TMFs [57–60, 64], while one is specific to the Indigenous health promotion context [62]. Four TMFs are action models [58–60, 64], that is they provide practical guidance about how to plan and carry out implementation activities [42]. The remaining two TMFs provide a description of equity-focused implementation but with less distinct steps or phases [57, 62].

All process TMFs identified have an equity focus. The EquIR aims to reduce or prevent inequities during implementation by providing a five-step, iterative process across the intervention design, implementation and evaluation pathway, each with an equity lens [58]. The framework authors provide an example of applying the EquIR to a programme for disadvantaged children in Bolivia and how the programme was adjusted based on equity considerations for each step of the EquIR [58]. The Transcreation framework aims to reduce health inequities by planning and delivering evidence-based interventions in a way appropriate for the local community context through a seven-step process [60]. This framework focuses on community partnership to reduce inequities in intervention adaptation and delivery [60]. The Transcreation framework has been used to develop a stress management programme for Latina cancer survivors by facilitating community engagement and programme adaptation [81].

The Collaborative Intervention Planning Framework provides a process for modifying interventions for new patient and provider populations with the goal of reducing health disparities [64, 82]. This framework applies Community-Based Participatory Research principles to the six-step Intervention Mapping process to support context-appropriate intervention adaption and implementation plan development [64, 82]. This framework has been used to adapt a care coordination and patient activation intervention for Hispanic people living with serious mental illness in order to increase its reach in this population [64, 82].

The I-RREACH tool aims to guide the process of identifying factors that influence implementation in low-resource settings (low- and middle-income countries and disadvantaged populations in high-income countries) by facilitating dialogue between the community and implementation team [59]. The tool outlines three phases (community profile, key informant perspective and community members perspective) where information is gathered to understand and assess the needs of the local community where the intervention is to be implemented [59]. Aboriginal populations in Canada and Tanzanian communities that participated in trialling the I-RREACH tool reported that it helped researchers understand their perspective, enhanced their understanding of the project and was culturally safe [59].

The ConNECT framework aims to address health inequities by applying five key principles across the implementation cycle from research to practice (Discovery, Development, Delivery and Dissemination): Integrating Context, Fostering a Norm of Inclusion, Ensuring Equitable Diffusion of Innovations, Harnessing Communication Technology and Prioritising Specialised Training [57]. This framework has not been operationalised.

The Indigenous Health Promotion Tool Implementation Model was developed to guide the successful implementation of health promotion tools in Indigenous primary care services, thereby reducing inequitable health outcomes for Indigenous peoples [62]. The model outlines four overlapping and inter-related processes guiding implementation: Engaging and Relating, Strengthening Capacity, Tailoring for Diversity in Programmes, Groups and Settings, and Developing and Using Evidence [62]. This model has not been operationalised.

The extent of systems-level focus in these process TMFs varies. All six process TMFs focus on the implementation context; the I-RREACH tool, Transcreation framework and Collaborative Intervention Planning Framework emphasise community engagement and partnership throughout the implementation planning process [59, 60, 82]. The EquIR specifies an assessment of the facilitators and barriers to equitable implementation, which, depending on the implementation context, could include micro-, meso- and macro-level factors [58]. The Indigenous Health Promotion Tool Implementation Model also includes micro-level influences in the domains of Reciprocity and Engaging and Relating [62].

### Evaluation frameworks

Three TMFs identified were evaluation frameworks (Table 3). RE-AIM is an established framework that was applied in an equity context [74], while the remaining two TMFs are equity-focused adaptations of established frameworks (RE-AIM and Proctor et al.’s framework) [11, 67].

RE-AIM is widely used to guide intervention and implementation planning and evaluation [83]. The RE-AIM framework includes five dimensions of implementation that can be assessed quantitatively and qualitatively [84]. While RE-AIM was not designed to be equity focused, it has been used to plan and evaluate an intervention delivered to a population that experiences ethnicity-related health inequities by identifying and addressing equity issues across the five dimensions [74]. RE-AIM has also been adapted to focus on sustainability and equity, with the goal of increasing health impact and equity [67]. RE-AIM primarily focuses on micro- and meso-level factors. Reach and Effectiveness of RE-AIM are individual-level dimensions, whereas Adoption and Implementation are provider/setting level dimensions; Maintenance can be both individual (e.g. long-term effectiveness or impact) and provider/setting (e.g. sustainability of the intervention or programme after implementation) level [84]. The adapted RE-AIM framework also includes explicit consideration of Dynamic Context and Culture, Costs, Resources and Capacity, and Health Equity across the implementation cycle [67].

Proctor et al.’s conceptual model of implementation research links implementation processes (intervention and implementation strategies) with outcomes (implementation, service and client) [85]. In the adapted framework, an equity lens is applied to selected elements with the aim of integrating implementation science and health inequity research (Table 2) [11, 85]. This includes focusing on intervention reach and implementation context from the beginning, developing implementation strategies to reduce inequities and assessing implementation outcomes from an equity perseptive [11]. The adaptated framework does not have a clear systems-level focus, although it does include a focus on Reach of the intervention and emphasises the context where implementation is to occur [11].

### Hybrid frameworks

One framework, the EQ-DI framework, was found to incorporate elements of determinant, process and evaluation TMFs and was, therefore, classified as a ‘hybrid’ framework (Table 3). EQ-DI is a high-level equity-focused framework that brings together elements of health equity and D&I science research to enhance each field [63]. In this framework, health equity sensitises D&I science by identifying, acknowledging and addressing the conditions in which inequities are created and perpetuated across multiple socio-ecological levels (individuals, relationships, community and system-level contexts) [63]. As a complement to this, D&I approaches in the framework operationalise health equity by providing tools, methods and approaches for planning and evaluation to disseminate and implement evidence-based health equity interventions [63]. The high-level nature of the D&I framework allows for other implementation science TMFs and health equity frameworks to be utilised within the framework (e.g. RE-AIM with an equity lens [63]).

## Discussion

This scoping review identified 15 implementation science TMFs, 12 of which had an equity focus that aimed to prevent or reduce inequities and three that were applied in an equity context; that is, to support intervention implementation in populations who experience ethnic health inequities. The TMFs were categorised and described, providing those implementing interventions with a resource to support appropriate TMF selection to facilitate equity-focused implementation.

Implementation science TMFs are used to understand the factors that support or hinder implementation, guide the implementation process (usually by describing steps or stages) and evaluate implementation outcomes, e.g. intervention reach, uptake, cost, appropriateness, sustainability [42]. The TMFs identified in this scoping review aligned with these broad aims while also focusing on achieving equity or reducing inequities. All but one TMF aligned with the classification system proposed by Nilsen, which describes five categories of TMFs according to their overarching aims and characteristics [42]. While TMFs can belong to more than one category and may have more than one purpose [42, 55], we considered the EQ-DI framework to be a hybrid of the determinant, process and evaluation TMFs [63]. Most TMFs were equity-focused, either explicitly (*n* = 8) or implicitly (*n* = 4), meaning that reducing or preventing inequities was the stated aim of the TMF or the study in which it was proposed. Additionally, most equity-focused TMFs were generic and could therefore be applied to a range of implementation contexts and target populations [11, 57–60, 63, 66, 67]. Two TMFs focused on Indigenous health [61, 62], and two TMFs were developed to address a particular health inequity [64, 65]. Three established TMFs were utilised to support the implementation of interventions in populations experiencing ethnic health inequities [68–73, 84]. While these three TMFs are not explicitly equity-focused, these studies illustrated how TMFs could be applied to equity contexts [68–73, 84]. In particular, the study by Glasgow and colleagues was an intentional and explicit equity-focused application of the RE-AIM framework [74]; the other two TMFs (CFIR and COM-B) had a more inherent equity focus due to the intervention’s target population [68–73].

Comparing the equity-focused and equity-applied TMFs within each category highlights similarities and differences in how equity and systems-level factors are incorporated. In the determinants category, the HEIF, CFIR, and integrated PRISM and SEM frameworks are comprehensive frameworks that identify implementation determinants across multiple levels of influence [65, 66, 78]. The HEIF also incorporates key equity domains derived from the Health Care Disparities Framework and the literature on health equity [66, 86]. In contrast, HPW focuses specifically on the determinants of appropriate and effective implementation for Indigenous populations but not broader factors that may facilitate or inhibit implementation [61]. Each determinant framework fully considered multi-level system influences; the HEIF, CFIR and integrated PRISM and SEM framework represent these across the multi-level domains of determinants, while HPW incorporates a systems-thinking domain [61, 65, 66, 78]. The equity-focused process models emphasise identifying community need, resources for implementation and making modifications to or adaptations of the intervention or implementation strategy to facilitate successful and equitable implementation [57–60, 62, 64]. Process models lend themselves less well to comprehensive systems-level thinking than determinant frameworks, likely due to their action-oriented nature, which necessitates a narrower, local-level focus.

In the evaluation category, the adaptated RE-AIM framework and the adaptation of Proctor et al.’s framework emphasise the application of an equity lens to implementation and evaluation activities [11, 67]. Evaluation frameworks tend to have a more comprehensive systems focus than process models, although macro-level factors are less well-represented than in determinant frameworks. While macro-level factors are typically more difficult to address or influence, intentional identification, which equity-focused TMFs can facilitate, is still important to enable implementation strategies to address barriers to equity at all levels.

With increasing recognition of the role implementation science can play in supporting and advancing health equity endeavours, the evidence base for the key factors that support equitable implementation is growing [27, 36, 37, 39, 40, 87], building on and incorporating approaches from health equity research [56, 88]. The inclusion of these key equity factors in TMFs ensures those undertaking implementation activities have guidance on how to do so in a way that will reduce or prevent inequities. For example, designing and selecting interventions with the implementation context in mind is recognised as an important factor in supporting equitable implementation as it focuses on who the intended target is and the particular challenges that different groups may face in accessing the intervention, e.g. due to cost, location, discrimination [11, 27]. Determinant frameworks such as the HEIF (or another determinant framework with an equity lens applied) can be used to systematically identify the barriers to equity and implementation [66]. These factors can then be addressed through design and implementation strategies that are tailored to the context [11, 27]. The Implementation Mapping process is an approach that has been developed to support the systematic planning or selection of implementation strategies for interventions [89]. In a case study of applying Implementation Mapping in a health equity context, Dickson et al. illustrated how the HEIF could be integrated into Implementation Mapping to ensure that explicit health equity determinants were explored and addressed through the process [90].

Another important equity factor is recognition of the role of structural racism in determining implementation and health equity outcomes [34–36]. Shelton et al. call for its inclusion in the implementation of TMFs and also encourage the use of multi-level approaches to address structural racism in implementation research and practice that involves minoritised ethnic groups [34]. This focus was not well represented in the TMFs identified in this review. However, recently an adaptation of the CFIR with a structural racism focus, utilised in evaluating the implementation of an equity intervention in a school setting, has been published [91]. Furthermore, based on user feedback, the CFIR has been updated to include subconstructs that reflect different aspects of equity that may influence implementation [92]. It includes caveats about the inclusion of equity experts and the use of equity-focused frameworks originating from outside implementation science to overcome the CFIR’s limitations.

Finally, developing trusting relationships and engaging with the community or group for whom the intervention is intended and other stakeholders is a key equity concept [27]. Participatory approaches vary in terms of the extent of stakeholder engagement, from maximal engagement (e.g. following the principles of community-based participatory research), to intermediate engagement (e.g. collaboration or consultation-based approaches) to minimal engagement (e.g. contractual approaches) [93]. Participatory approaches can be utilised across a range of implementation research activities, including selecting the health issue to be addressed and/or the intervention, developing community research capability and capacity, and dissemination activities [93]. Recently, a community-based participatory research model has been applied as an implementation framework to support community-academic research partnerships [94]. Relationship development and community engagement are well represented in the process models identified in this review, which encourage this action step early in the implementation process [57–60]; interestingly, the EquIR did not make community engagement explicit in the programme planning phase [58].

Evaluating implementation outcomes is a crucial part of the implementation process to determine the success or failure of the implementation pathway for achieving the desired outcomes. Applying an equity lens ensures that the implementation pathway can be evaluated with respect to how well inequities are likely to be prevented or reduced and how this relates to intervention effectiveness [11]. The EquIR provides an example of how established implementation outcomes (as developed by Proctor et al. [85]) can be viewed with an equity focus [58].

### Strengths

This scoping review identifies and describes existing equity-focused implementation science TMFs, as well as general TMFs operationalised with an equity focus, with a particular interest in those involved in reducing or preventing ethnic health inequities. We included literature from a wide range of sources and this was reviewed by experts in health equity, implementation science and Māori health to ensure that any gaps were addressed. The TMFs were categorised according to a well-established taxonomy [42]. A further strength is the inclusion of examples of how TMFs were operationalised to illustrate their practical application. These findings also complement the D&I model database special topics section on health equity that also identifies TMFs used in a health equity context (https://dissemination-implementation.org/special-topics/health-equity/).

### Limitations

There are some limitations to this review. We limited our search to two databases of the peer-reviewed literature, meaning other potentially relevant TMFs and examples of their operationalisation may not have been identified. Similarly, due to our interest in ethnic health inequities and healthcare interventions, we may have missed examples where TMFs were operationalised in other populations or settings. We also note the limitations of terminology, with TMFs being described in ways that are inconsistent with definitions or being used interchangeably due to a lack of agreement within the discipline of implementation science about where TMFs ‘best fit’, which makes viewing these through a health equity lens even more challenging.

### Future directions

There is significant scope for future research to consider TMFs and implementation studies utilised in non-healthcare settings to determine whether valuable learnings could be applied from these other contexts. Several TMFs in this review had not yet been operationalised, and most TMFs had not been operationalised in more than one or two studies. Future application of these TMFs would be useful to further an understanding of how relevant they are in supporting equity in implementation endeavours, as well as guiding researchers and practitioners about how to select a TMF to best fit equity-focused research questions.

## Conclusion

This scoping review identifies and summarises the equity-focused implementation science TMFs available to support health researchers, clinicians, funders and other decision-makers to undertake equity-focused implementation. It also identifies general TMFs that have been operationalised with an equity focus. By collating the information on the growing number of equity-focused and equity-applied TMFs, prospective users may be able to identify and select the most appropriate TMF to guide implementation research and utilise the examples of how these TMFs have been operationalised.

### Supplementary Information

**Additional file 1. **Preferred Reporting Items for Systematic reviews and Meta-Analyses extension for Scoping Reviews (PRISMA-ScR) Checklist

**Additional file 2. **Database search strategies. 

**Additional file 3.  **Grey literature search strategy

**Additional file 4: Table S1. **Implementation science TMF aims, categories and descriptions [42].

## Data Availability

Not applicable.

## Abbreviations

CFIR: Consolidated Framework for Implementation Research
COM-B: Capability, Opportunity, Motivation and Behaviour
D&I: Dissemination and Implementation
EquIR: Equity-based Framework for Implementation Research
HEIF: Health Equity Implementation Framework
HPW: He Pikinga Waiora
I-RREACH: Intervention and Research Readiness Engagement and Assessment of Community Health Care
PRISM: Practical, Robust Implementation and Sustainability Model
SEM: Socio-Ecological Model
PRISMA-ScR: Preferred Reporting Items for Systematic Reviews and Meta-Analyses extension for Scoping Review
RE-AIM: Reach, Effectiveness, Adoption, Implementation and Maintenance
TMF: Theory, Model and Framework
