# Supplementary material for: A scoping review of equity-focused implementation theories, models and frameworks in healthcare and their application in addressing ethnicity-related health inequities
Source: Implement Sci. 2023 Oct 16;18:51. doi: 10.1186/s13012-023-01304-0 (PMC10578009; doi:10.1186/s13012-023-01304-0)
Supplement: Supplementary file 2 — Additional file 2. Database search strategies. [file 13012_2023_1304_MOESM2_ESM.docx]

**Additional file 2.** Database search strategies.

**MEDLINE search strategy**

| Search terms | Results |
| --- | --- |
| 1. (implementation science or implementation framework or implementation research or implementation process or implementation effectiveness or knowledge transfer or knowledge exchange or knowledge translation).af. | 17582 |
| 1. (framework* or theor* or model* or checklist* or classifi* or categor* or concept* or tool or protocol).af. | 6273049 |
| 1. 1 and 2 | 10360 |
| 1. (health intervention or health care or healthcare or evidence-based intervention or evidence-based practice or health service*).af. | 1615142 |
| 1. 3 and 4 | 6786 |
| 1. limit 5 to (english language and humans and yr="2011 -Current") | 6110 |
| 1. (equity or health equity or inequal* or health inequal* or disparit* or health diparit* or inequit* or health inequit*).af. | 152517 |
| 1. 6 and 7 | 486 |

**CINAHL search strategy**

| Search terms | Results |
| --- | --- |
| 1. implementation science or implementation framework or implementation research or implementation process or implementation effectiveness or knowledge transfer or knowledge exchange or knowledge translation | 20,885 |
| 1. framework* or theor* or model* or checklist* or classifi* or categor* or concept* or tool or protocol | 1,710,935 |
| 1. S1 AND S2 | 13,286 |
| 1. health intervention or health care or healthcare or evidence-based intervention or evidence-based practice or health service* | 1,011,435 |
| 1. S3 AND S4; Limiters - Published Date: 20110101-20220131 ; English Language; Human | 3,807 |
| 1. equity or health equity or inequal* or health inequal* or disparit* or health diparit* or inequit* or health inequit* | 82,265 |
| 1. S5 AND S6 | 191 |

**D&I database search strategy**

| Search criteria: |
| --- |
| 1. D And/Or I: Implementation |
| 1. Socio-Ecological levels: All |
| 1. Constructs: Health Equity |

**Results:** 4

| **ID** | **Name** | **D_I** | **Construct Flexibility** | **Field of Origin** | **Citation** | **Citations Case Exmpl** | **# Times Cited** | **PractnerResearcher** |
| --- | --- | --- | --- | --- | --- | --- | --- | --- |
| 131 | Transcreation Framework for Community-engaged Behavioral Interventions to Reduce Health Disparities | I-Only | 1 | Behavioral Health Services | Nápoles, A.M., Stewart, A.L. Transcreation: an implementation science framework for community-engaged behavioral interventions to reduce health disparities. BMC Health Serv Res 18, 710 (2018). https://doi.org/10.1186/s12913-018-3521-z | Santoyo-Olsson J, Stewart AL, Samayoa C, Palomino H, Urias A, Gonzalez N, et al. (2019) Translating a stress management intervention for rural Latina breast cancer survivors: The Nuevo Amanecer-II. PLoS ONE 14(10): e0224068. https://doi.org/10.1371/journal.pone.0224068  Nápoles, AM, Santoyo-Olsson, J, Stewart, AL, et al. Nuevo Amanecer-II: Results of a randomized controlled trial of a community-based participatory, peer-delivered stress management intervention for rural Latina breast cancer survivors. Psycho-Oncology. 2020; 29: 1802 –1814. https://doi.org/10.1002/pon.5481 | 19 | R+P |
| 114 | Health Equity Implementation Framework | I-Only | 5 | Public Health; Medicine | Woodward, E. N., Matthieu, M. M., Uchendu, U. S., Rogal, S., & Kirchner, J. E. (2019). The health equity implementation framework: proposal and preliminary study of hepatitis C virus treatment. Implement Sci, 14(1), 26. doi:10.1186/s13012-019-0861-y | | 4 | Researcher |
| 122 | EQ-DI Framework | D=I | 3 | Public Health | Yousefi Nooraie, R., Kwan, B., Cohn, E., AuYoung, M., Clarke Roberts, M., Adsul, P., & Shelton, R. (2020). Advancing health equity through CTSA programs: Opportunities for interaction between health equity, dissemination and implementation, and translational science. Journal of Clinical and Translational Science, 4(3), 168-175. doi:10.1017/cts.2020.10 | Gordon, EJ, Romo, E, Amórtegui, D, et al. Implementing culturally competent transplant care and implications for reducing health disparities: A prospective qualitative study. Health Expect. 2020; 00: 1 –15. https://doi.org/10.1111/hex.13124 | 2 | Researcher and Practitioner |
| 113 | conNECT Framework | I>D | 3 | Nursing | Alcaraz KI, Sly J, Ashing K, Fleisher L, Gil-Rivas V, Ford S, Yi JC, Lu Q, Meade CD, Menon U, Gwede CK. The ConNECT Framework: a model for advancing behavioral medicine science and practice to foster health equity. J Behav Med. 2017 Feb;40(1):23-38. doi: 10.1007/s10865-016-9780-4. | Ashing KT, Soto-Perez-de-Celis E. Disparities within a disparity: Global health and health equity in geriatric oncology. J Geriatr Oncol. 2020 Mar;11(2):200-202. doi: 10.1016/j.jgo.2019.06.007  Menon, U., Ashing, K., Chang, M. W., Christy, S. M., Friberg-Felsted, K., Rivas, V. G., ... & Wang, M. (2019). Application of the ConNECT framework to precision health and health disparities. Nursing Research, 68(2), 99-109. | 1 | Researcher |
