# Supplementary material for: A scoping review of equity-focused implementation theories, models and frameworks in healthcare and their application in addressing ethnicity-related health inequities
Source: Implement Sci. 2023 Oct 16;18:51. doi: 10.1186/s13012-023-01304-0 (PMC10578009; doi:10.1186/s13012-023-01304-0)
Supplement: Supplementary file 3 — Additional file 3. Grey literature search strategy [file 13012_2023_1304_MOESM3_ESM.docx]

**Additional file 3**

**Grey literature search strategy**

The grey literature search was conducted with Google. The search was limited to literature published on governmental and non-governmental organisation websites in New Zealand between 1 January 2011 and the present (final search executed 14 April 2022).

**Google search strategy**

| Search criteria: |
| --- |
| “health” AND “equity” AND “implementation” AND “framework or model or theory” |
