# Supplementary material for: A scoping review of equity-focused implementation theories, models and frameworks in healthcare and their application in addressing ethnicity-related health inequities
Source: Implement Sci. 2023 Oct 16;18:51. doi: 10.1186/s13012-023-01304-0 (PMC10578009; doi:10.1186/s13012-023-01304-0)
Supplement: Supplementary file 4 — Additional file 4: Table S1. Implementation science TMF aims, categories and descriptions [42]. [file 13012_2023_1304_MOESM4_ESM.docx]

**Additional file 4**

**Table S1.** Implementation science TMF aims, categories and descriptions [42].

| **Overarching aim** | **TMF category** | **Description** |
| --- | --- | --- |
| Understand and/or explain what influences implementation outcomes | Determinant framework | Specify types/classes/domains of determinants which act as barriers and enablers that influence implementation outcomes. |
|  | Classic theories | Theories that originate from fields external to implementation science that can be applied to provide understanding and/or explanation of aspects of implementation. |
|  | Implementation theories | Theories developed by implementation researchers to provide understanding and/or explanation of aspects of implementation. |
| Describe and/or guide the process of translating research into practice | Process models | Specify steps (stages, phases) in the process of translating research into practice, including the implementation and use of research. The aim of process models is to describe and/or guide (i.e. the action-type models) the process of translating research into practice. |
| Evaluate implementation | Evaluation frameworks | Specify aspects of implementation that could be evaluated to determine implementation success. |
